# Supplementary material for: The First Poly(A) Polymerase from Alphaproteobacteria
Source: Int J Mol Sci. 2026 Mar 7;27(5):2467. doi: 10.3390/ijms27052467 (PMC12985599; doi:10.3390/ijms27052467)
Supplement: Supplementary file 1 [file ijms-27-02467-s001.zip › ijms-4054096-supplementary.pdf]

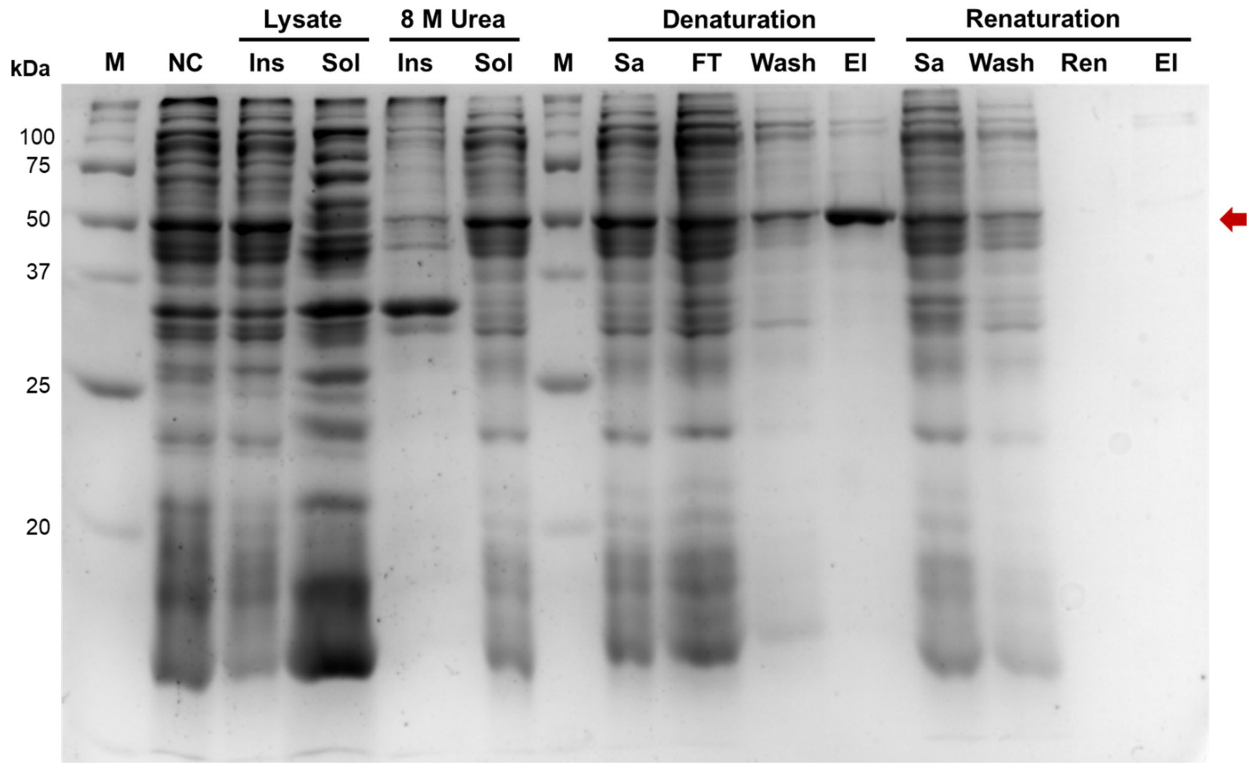

Supplementary Figure S1. Purification of Mli PAP on Ni-charged IMAC resin. The enzyme was expressed in *E. coli* strain BL21 (DE3) pLysS. M — Precision Plus Protein standards (Bio-Rad, Hercules, CA, USA), NC — *E. coli* lysate before expression of Mli PAP, Lysate — lysate after expression of Mli PAP, Ins — insoluble fraction, Sol — soluble protein fraction, 8 M Urea — inclusion bodies solubilized by 8 M urea, Denaturation — purification under denaturation conditions, Renaturation — purification coupled with renaturation on a column, Sa — sample, applied on a Ni-charged IMAC resin, FT — flow-throw, Wash — column wash, Ren — renaturation on a column by a reverse urea gradient (8–0 M), El — eluate. Mli PAP is marked by a red arrow.

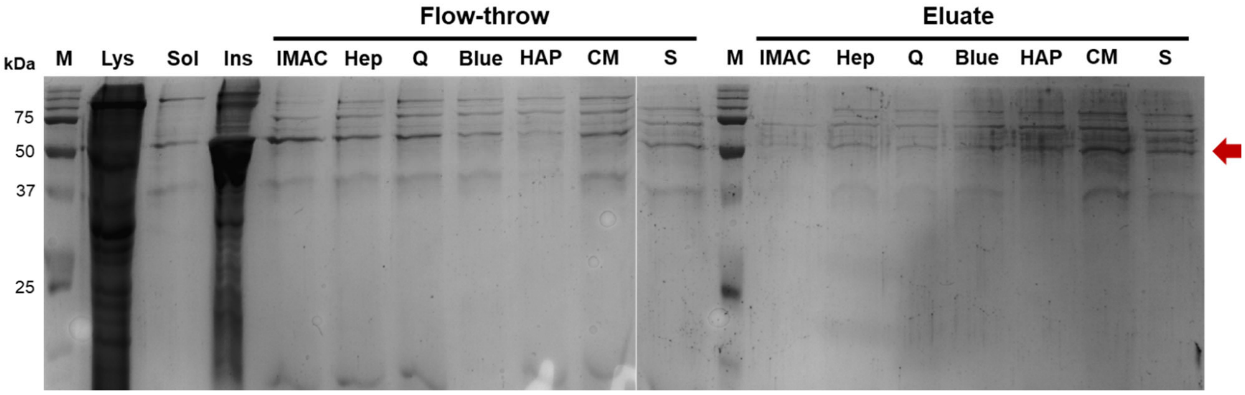

Supplementary Figure S2. Purification of Mli PAP on Ni-charged IMAC resin. The enzyme was expressed in *E. coli* strain BL21 (DE3) pLysS. M — Precision Plus Protein standards (Bio-Rad, Hercules, CA, USA), Lys — lysate after expression of Mli PAP, Sol — soluble protein fraction, Ins — insoluble fraction, Flow-throw — flow-throw, Eluate — eluate, IMAC — Ni-charged IMAC resin,Hep — Heparin SepFast, Q — Macro-Prep HighQ Media, Blue — Affi-Gel Blue Media, HAP — CHT Ceramic Hydroxylapatite Type II, CM — Macro-Prep CM Support, S — Macro-Prep HighS Support. Mli PAP is marked by a red arrow.
